# Exploring the factors influencing the use of health services by people with diabetes in Northwest China: an example from Gansu Province

**DOI:** 10.1186/s41043-023-00402-5

**Published:** 2023-07-07

**Authors:** Ying Dang, Yinan Yang, Shuting Cao, Jia Zhang, Xiao Wang, Jie Lu, Qijun Liang, Xiaobin Hu

**Affiliations:** 1 Department of Epidemiology and Statistics, School of Public Health, Lanzhou University, Lanzhou, Gansu China; 2 Department of Pediatric Cardiology, Lanzhou University Second Hospital, Lanzhou, Gansu China; 3 Health Statistics Information Center of Gansu Province, Lanzhou, Gansu Province China; 4 Gansu Medical Insurance Service Centre, Lanzhou, Gansu Province China

**Keywords:** Diabetes, Health service utilization, Influence factors, Random forest

## Abstract

**Background:**

Diabetes is associated with high morbidity, mortality and quality-of-life impairment in patients. In China, the number of people suffering from diabetes ranks first in the world. Gansu Province is located in northwest China and is an economically underdeveloped region of China. By analyzing the level of health service utilization of people with diabetes in Gansu Province, the degree of equity in health service utilization and its influencing factors were studied to provide scientific data to support the promotion of health equity for people with diabetes and the introduction of relevant policies by relevant authorities.

**Methods:**

A sample of 282 people with diabetes who were 15 years old and above was chosen by multi-stage stratified sampling method. A structured questionnaire survey was conducted via face-to-face interviews. Random forest and logistic regression analysis were used to demonstrate the effects of the explanatory variables on health seeking behaviors from predisposing, enabling and need variables. The concentration index was used to indicate the equity of health service utilization across households of different economic levels.

**Results:**

The outpatient rate for the diabetic population surveyed was 92.91%, with 99.87% of urban patients, higher than the 90.39% of rural patients. The average number of hospital days per person was 3.18 days, with 5.03 days per person in urban areas, which was higher than the 2.51 days per person in rural areas. The study showed that the factors most likely to influence patients to seek outpatient services were frequency of taking diabetic medication, whether or not they were contracted to a household doctor, and living environment; the top three factors most likely to influence patients with diabetes to seek inpatient services were number of non-communicable chronic disease, self-assessment of health status, medical insurance. The concentration index for outpatient service utilization and inpatient service utilization were − 0.241 and 0.107, respectively, indicating that outpatient services were concentrated on patients at lower income levels and patients at higher income levels tended to favor inpatient services.

**Conclusion:**

This study found that the low level of health care resources available to people with diabetes, whose health status is suboptimal, makes it difficult to meet their health needs. Patients' health conditions, comorbidities of people with diabetes, and the level of protection were still important factors that hindered the use of health services. It is necessary to promote the rational use of health services by diabetic patients and further improve the corresponding policies to achieve the goal of chronic disease prevention and control in “Health China 2030”.

## Background

Diabetes Mellitus (DM) is a chronic disease associated with high morbidity, mortality and quality of life impairment in patients [1]. The International Diabetes Federation (IDF) has released the latest IDF Diabetes Atlas 2021 (10th edition), which shows that approximately 537 million people will be living with diabetes in 2021 [2]. In China, the number of people aged 20–79 with diabetes ranks first in the world at 116 million in 2021, and diabetes has become a major public health problem in China. People with diabetes are often comorbid with other non-communicable chronic diseases (NCDs) and a national survey study suggests that about 2/3 of Chinese people with diabetes are comorbid with other NCDs [3]. After a long period of exploration, China has established a multi-level medical security system with basic medical insurance for urban workers and urban residents as the mainstay, supplemented by large subsidies and commercial medical insurance, etc., and backed by social medical assistance [4]. However, with the increasing prevalence of diabetes and the increasing financial burden of the disease on patients, the current level of medical coverage for diabetes is unable to meet the current demand for health services for diabetics [5].

As one of the important livelihood issues, health service utilization equity is one of the important signs of social equity and one of the important bases for the formulation of health care policy [6]. Studying and improving the equity in health services and encouraging health care reforms that promote the public good and increase equity is an important task in building a harmonious society and ensuring social equity. However, with socio-economic development, the gap between the rich and the poor continues to widen, regional development becomes more uneven and the equity of health services is seriously challenged [7, 8]. A study showed that 84.4% of older people visited a health facility in the past year, and determines that monthly household income, chronic conditions, medication use by older people, and self-rated health status were strongly associated with health service utilization [9]. Another study showed that the rate of health service-seeking behavior among migrants (46.8%) was lower than among residents with “Hukou” (62.6%) [10]. In addition, it has been suggested that people with intellectual and developmental disabilities (IDD) often have complex health needs and are heavy users of health services [11].

Much of the current research on health service utilization and the economic risk of disease has focused on population-wide studies, with less research on the health service utilization of patients with specific NCDs and the economic risk of disease they pose. Gansu Province, located in northwest China and the upper reaches of the Yellow River, is an economically underdeveloped region of China and also home to a large number of minorities. This study aims to investigate the level of health service utilization, influencing factors, and equity of diabetic patients in Gansu Province, to promote health equity for diabetic patients and to provide scientific data to support the relevant departments in introducing relevant policies.

## Methods

### Data source and participants

The Chinese 6th National Health and Services Survey (NHSS) has been conducted nationwide every five years since 1993 [12]. Data for this study were obtained from the Gansu Provincial Health Services Household Survey from August to October 2018. The study was led by the Gansu Provincial Health and Health Commission, and surveyors conducted face-to-face surveys in residents' homes using tablet computers (pads). This survey adopted multi-stage random sampling of the whole group, following the principle of economic validity while ensuring the representativeness of the sample and assessing the whole population through the sample. First, five districts/counties were selected (Yuzhong, Jingtai, Lintan, Maiji, and Ganzhou). Second, all towns (streets) in each selected district (counties) were divided into five levels, and one town or street is randomly selected at each level. Third, two villages (residential committees) were randomly selected from each selected town or street.

### Analytic framework

**Fig. 1:**
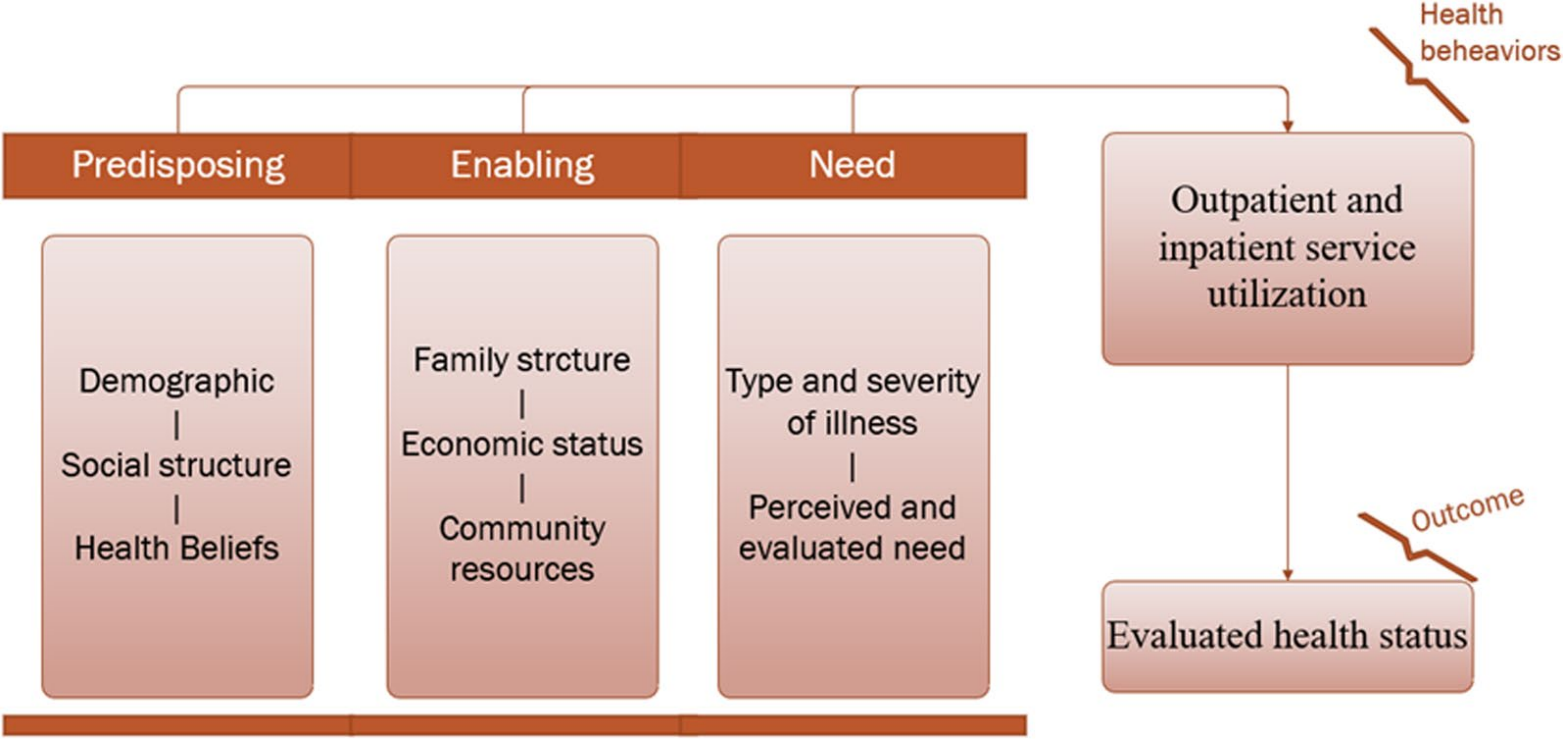
The simplified Anderson services utilization model

Anderson's behavior model, created in 1968 by Professor Ronald M. Andersen at the University of California School of Public Health in Los Angeles, is the most commonly adopted model for the study and analysis of health service utilization and is widely used in health system evaluation and health service research [13]. Current international health service utilization studies focusing on subpopulations include low-income populations, older adults, and people with NCDs [14]. We used the simplified Anderson Health Services Model to analyze the factors influencing outpatient service utilization and inpatient service utilization. We divided the factors affecting health service utilization into three categories: predisposing factors, enabling factors, and need factors. Predisposing factors are characteristics that determine or motivate people to use health services should the need arise, including demographic characteristics, social structure, health beliefs, etc. Enabling factors refer to an individual's ability and resources to access health services, including household income, health insurance, community health resources, etc. Need factors include subjective judgments of symptoms and assessments of one's illness by qualified medical practitioners, etc. (See Fig. 1). This study was analyzed by drawing on measures commonly used in relevant studies to describe health service utilization, using outpatient rate (see Eq. 1), inpatient rate (see Eq. 2), and days in the hospital as indicators to evaluate the level of health service utilization of people with diabetes.

1 $${\text{Outpatient}}\;{{\rm rate}} = \frac{{{{\rm number}}\;{{\rm of}}\;{{\rm visits}}\;{{\rm to}}\;{{\rm a}}\;{{\rm health}}\;{{\rm facility}}\;{{\rm for}}\;{{\rm illness}}\;{{\rm or}}\;{{\rm injury}}\;{{\rm in}}\;{{\rm the}}\;{{\rm two}}\;{{\rm weeks}}\;{{\rm prior}}\;{{\rm to}}\;{{\rm the}}\;{{\rm survey}}}}{{{{\rm number}}\;{{\rm of}}\;{{\rm people}}\;{{\rm surveyed}} \times {1}00\% }}$$

2 $${\text{Inpatient}\,{\rm rate}} = \frac{{{\text{actual}}\;{{\rm number}}\;{{\rm of}}\;{{\rm hospitalizations}}\;{{\rm in}}\;{{\rm the}}\;{{\rm year}}\;{{\rm prior}}\;{{\rm to}}\;{{\rm the}}\;{{\rm survey}}}}{{{{\rm number}}\;{{\rm of}}\;{{\rm people}}\;{{\rm surveyed}} \times {1}00\% }}$$

### Random forest model construction

The use of artificial intelligence (AI) and machine learning (ML) algorithms is gaining increasing recognition in various fields and industries, including the healthcare industry [15]. Random forests have many advantages over traditional methods of analyzing influencing factors such as logistic regression. Firstly, logistic regression requires a certain sample size, the more influencing factors are analyzed, the higher the sample size requirement; while random forest not only has no limitation on the sample size but can also analyze data where the number of influencing factors is much larger than the sample size. Second, random forest developed several decision trees using a subset of independently obtained random variables with replacement from the original dataset so that overfitting can be avoided. Since random forest uses bootstrap resampling technique in constructing each decision tree, it uses different training samples to construct different decision trees. This avoids overdependence of the decision tree on the training data, which can lead to overfitting. This advantage is especially obvious for small sample data [16]. An important feature of random forest is the inbuilt feature importance function, which allows the importance of explanatory variables in the development of the outcome to be evaluated [17]. In this study, the dataset was divided into two parts to test the effectiveness of the model and to test the generalizability of the model. A training set containing 70% of the data and a test set containing 30% of the data was used to construct the data for the model and to identify the best model, respectively. The outcome variable in this study was whether or not people with diabetes used health services, which was the ultimate aim of the decision classification. The explanatory variables were statistically significant in univariate terms. The average Gini index reduction method, focuses on the impurity of each variable in directly reducing the nodes of the decision tree and thus derives its categorical importance, which provides a theoretical basis for comparison of importance between variables. So we used the average decrease Gini index to measure the importance of the variables in the random forest prediction model to identify relevant factors that had a significant impact on patient health service utilization. The greater the average decline in the Gini coefficient, the more important the explanatory variables were to the classification.

### Concentration index

In households with diabetic patients, the utilization of health services varied with the economic level of the household [18]. To indicate the extent of such variation, Wagstaff proposed the concentration index (CI), a quantitative criterion with values between − 1 and 1 [19]. When the CI > 0, it indicates that patients with a high economic level seek health services more frequently, while when the CI < 0, it implies the opposite. The CI was calculated in Eq. (3), where h_i_ represents the fractional rank of individual i in relation to health status, Ri represents the fractional rank of individual i in relation to economic status, and *n* represents the sample size.

3 $${\text{CI}}\left( {h|y} \right) = \frac{{2{\text{cov}} \left( {h_{i} ,R_{i} } \right)}}{{\overline{h}}} = \frac{1}{n}\mathop \sum \limits_{i = 1}^{n} \left[ {\frac{{h_{i} }}{{\overline{h}}}\left( {2R_{i} - 1} \right)} \right]$$

As the indicator chosen for this study was whether health services were sought, it was a dichotomous variable. Therefore, a modified formula proposed by Wagstaff was used in this study to calculate the CI [20]. In Eq. (4), *h*_max_ and *h*_min_ represent the maximum and minimum values of the h-index in the sample, respectively.

4 $${\text{Wagstaff.CI}}\left( {h|y} \right) = \frac{1}{n}\left[ {\frac{{\left( {h^{\max } - h^{\min } } \right)h_{i} }}{{\left( {h^{\max } - \overline{h}} \right)\left( {\overline{h} - h^{\min } } \right)}}} \right]\left( {2R_{i} - 1} \right)$$

### Statistic analysis

Statistical analysis was performed with R software, and CI was calculated using Stata software. Statistical descriptions of the count data were made using percentages and the *χ*^2^ test was used for one-way analysis. The study data from 282 people with diabetes were randomly divided into a training set and a test set in the ratio of 7:3. Variables which indicated significant differences (*P* < 0.05) in utilization of service were retained and brought into the data of the training set, modeled using the random forest algorithm to explore the factors influencing health service utilization, and the variables were ranked in order of importance and then validated in the test set. We used the random forest model and the average Gini index reduction method to assess the contribution of each predictor variable to the classification accuracy of the model and thus determine the relative importance of the variables. The average Gini index reduction method assesses the importance of each variable based on its contribution to the reduction of the Gini index of a node during the construction of the decision tree, which provides an intuitive picture of the contribution of the variable to the improvement of classification accuracy. Also, variables with *P* < 0.05 in the univariate analysis were subjected to binary logistic regression analysis to obtain relative risk levels of the influencing factors.

## Results

### Basic characteristics of people with diabetes

There were 143 (50.71%) males and 139 (49.30%) females with diabetes in this survey (See Table 1). The ethnic group was Han Chinese with 260 patients (92.20%) and other ethnic groups with 22 patients (7.80%). The majority of patients (95.74%) were aged 45 years an d above. The majority of the people with diabetes (83.30%) were suffering from type 2. The number of patients with combined hypertension was 151 (53.55%).

**Table 1:** Basic characteristics of diabetic patients

Variables	Number (*n*)	Percent (%)
*Sex*		
Male	143	50.71
Female	139	49.30
*Ethnicity*		
Han	260	92.20
Minority	22	7.80
*Location*		
Urban	75	26.60
Rural	207	73.40
*Age group*		
15-	12	4.26
45-	140	49.65
65-	130	46.10
*Marital status*		
Married	235	83.33
Unmarried	47	16.67
*Education*		
Primary school	75	26.60
Middle school	82	29.08
High school	73	25.89
University	52	18.44
*Type of diabetes*		
1	9	3.20
2	235	83.30
Unknown	38	13.50
*Frequency of drugs*		
Always	215	76.20
Sometimes	49	17.40
Never	18	6.40
*Type of CHE*		
1	102	36.17
2	125	44.33
≥ 3	55	19.50
*Hypertension*		
Yes	151	53.55
No	131	46.45

### The condition of health service of people with diabetes

The outpatient rate for the diabetic population in this survey was 92.91%, with 99.87% for urban patients, significantly lower than the 99.87% for rural patients; the outpatient rate was 99.30% for males and 86.33% for females; the outpatient rate for patients aged 65 years and above (96.30%) was significantly higher than for those in the 15 year old group. The average number of hospital days per person in the diabetic population surveyed was 3.18 days, with 5.03 days per person in urban areas, higher than the 2.51 days per person in rural areas; the number of hospital days per person for men (3.68d) and women (2.66d) was comparable. Among the diabetic population surveyed, the inpatient rate was 24.82%, with 36.00% for those living in urban areas and 20.77% for those living in rural areas; the inpatient rate for men was 27.27%, less than the 22.30% rate for women; the inpatient rate for patients in the 65-year-old group (26.92%) was higher than that for patients in the 15-year-old group (16.67%) (See Table 2).

**Table 2:** Health service utilization of people with diabetes in Gansu Province

	Outpatient rate (%)	Inpatient days (day)	Inpatient rate (%)
*Local*			
Urban	99.87	5.03	36.00
Rural	90.39	2.51	20.77
*Sex*			
Male	99.30	3.68	27.27
Female	86.33	2.66	22.30
*Age*			
15-	90.06	1.67	16.67
45-	90.00	3.24	23.57
65-	96.30	3.25	26.92
Total	92.91	3.18	24.82

### Factors influencing health services among people with diabetes

In order to reduce the error in the multi-factor regression results and random forest results, variables that were significant in the one-way analysis were included in the random forest analysis and binomial logistic regression for the analysis of influencing factors in this study. The selected independent variables were first classified according to the simplified Anderson Health Services Model and subjected to univariate analysis using whether they used outpatient services and whether they used inpatient services as the dependent variables (See Table 3). The result showed that ethnicity, household doctor and physical examination were the predisposing factors that influenced whether patients used the outpatient services (*P* < 0.05). However there was no statistical difference in the predisposing factors that influenced whether patients used the inpatient services. The enabling factors influencing the use of outpatient services included location and distance to the nearest medical facility, while the only enabling factor influencing the use of inpatient services was location. Need factors influencing whether patients used outpatient services included anxiety or depression, and frequency of diabetes medication use. Need factors that affected whether patients used inpatient services included self-assessment of health status, physical pain, anxiety or depression, number of NCDs.

**Table 3:** Single factor analysis influencing health service utilization

	Whether to use outpatient services				Whether to use inpatient services			
	*n*	%	*χ*^2^	*P*	*n*	%	*χ*^2^	*P*
*Proposing factors*								
Sex								
Male	65	45.45	1.261	0.261	39	27.27	0.933	0.334
Female	54	38.85			31	22.30		
Ethnicity								
Han	115	44.23	5.643	0.017	68	26.15	3.165	0.075
Minority	4	18.18			2	9.09		
Age group								
15-	1	8.33	5.895	0.052	2	16.67	0.853	0.653
45-	61	43.57			33	23.57		
65-	57	43.85			35	26.92		
Household doctor								
Yes	96	45.28	7.256	0.027	55	25.94	0.824	0.662
No, but understand	4	16.67			6	25.00		
No, but do not understand	19	41.30			9	19.57		
Physical examination								
Yes	95	49.22	12.370	< 0.001	53	27.46	2.281	1.131
No	24	26.97			17	19.10		
*Enabling factors*								
Location								
Urban	16	21.33	18.237	< 0.001	27	36.00	6.840	0.009
Rural	103	49.76			43	20.77		
Health care insurance								
UEBMI^a^	85	40.28	4.635	0.099	45	21.33	6.256	0.044
Other	29	44.62			22	33.85		
None	5	83.33			3	50.00		
Distance								
≤ 1 km	70	37.63	4.666	0.031	43	23.12	0.851	0.356
> 1 km	49	51.04			27	28.13		
Time								
≤ 15 min	92	40.17	2.046	0.153	55	24.02	0.423	0.515
> 15 min	27	50.94			15	28.30		
*Need factors*								
Self-assessment of health status^b^								
Quintile 1	4	50.00	5.713	0.222	3	37.50	12.578	0.014
Quintile 2	6	31.58			7	36.84		
Quintile 3	49	41.88			38	32.48		
Quintile 4	57	46.72			21	17.21		
Quintile 5	3	1875			1	6.25		
Physical pain								
No problems	65	38.46	2.415	0.120	29	17.16	13.271	< 0.001
Medium or serious problems	54	47.79			41	36.28		
Anxiety or depression								
No problems	88	96.21	6.334	0.012	48	21.05	9.069	0.003
Medium or serious problems	31	57.41			22	40.74		
Number of NCDs^c^								
1	36	35.29	3.322	0.190	16	15.69	17.285	< 0.001
2	59	47.20			29	23.20		
3 or more	24	43.64			25	45.45		
Type of diabetes								
1	1	11.11	3.725	0.155	2	22.22	2.001	0.368
2	101	42.98			62	26.38		
No clear	17	44.74			6	15.79		
Frequency of diabetes medication use								
Regular dosing	103	47.91	12.615	0.002	56	26.05	0.962	0.618
Occasionally	13	26.53			11	22.45		
Never	3	16.67			3	16.67		

^a^UEBMI refers to Urban Employees Basic Medical Insurance

^b^Quintile 1 is the poorest and Quintile 5 is the wealthiest

^c^1 means patients with diabetes only; 2 means people with diabetes suffer from another chronic disease; 3 or more means people with diabetes with two or more chronic diseases

**Fig. 2:**
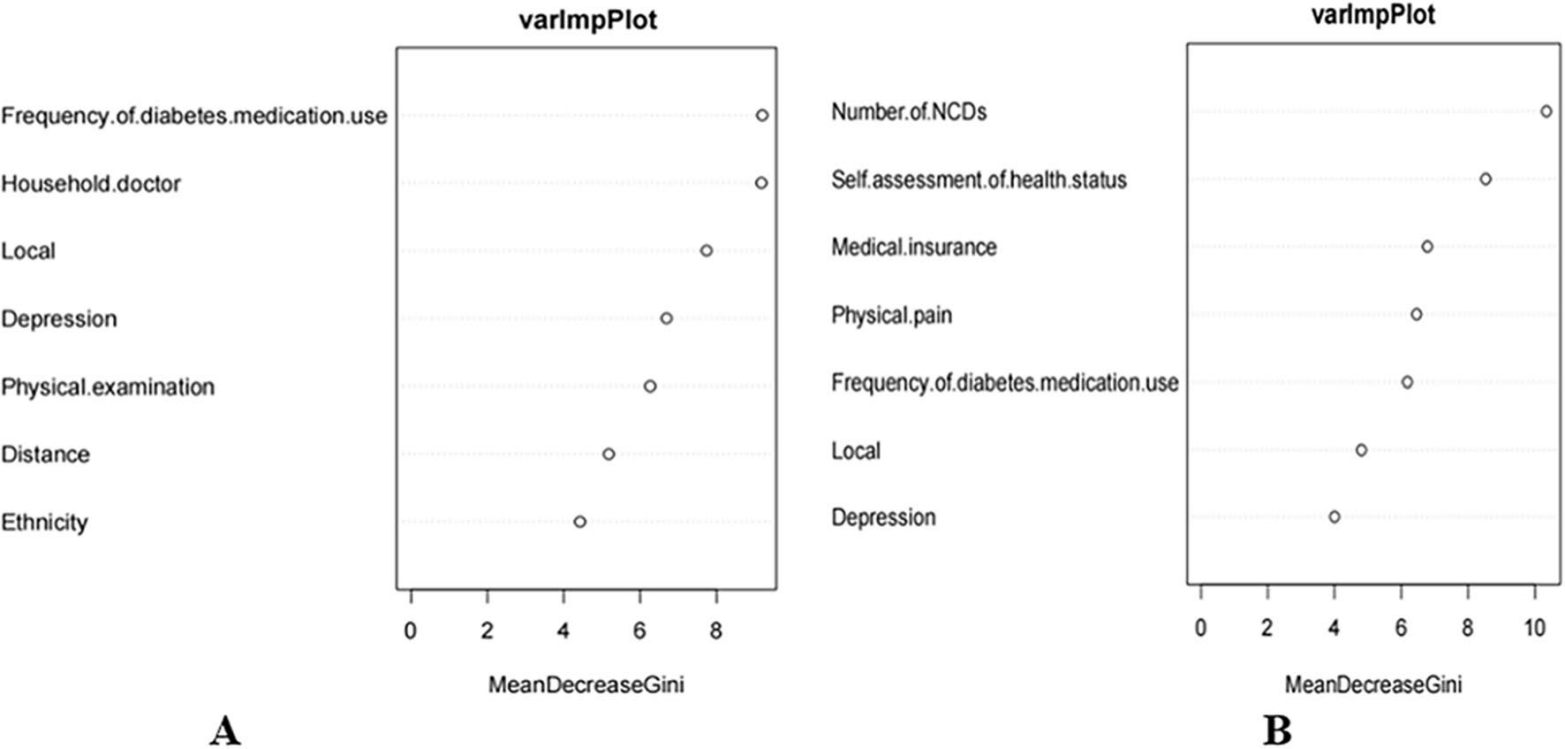
Feature importance for health service utilization: feature importance for outpatient service utilization (**A**), feature importance for inpatient service utilization (**B**)

The contribution of each variable to the classification accuracy of the model was assessed by means of a random forest model and a mean Gini reduction index. We plotted the importance of factors for health services through a random forest model (See Fig. 2). After training the algorithm and optimizing the model, the variable feature importance scores for outpatient service utilization and inpatient service utilization were plotted. The y-axis represented the explanatory variables, while the x-axis represented the feature importance score. Higher scoring characteristics were considered as variables that were more critical in predicting the importance of health service utilization. Figure 2a demonstrates the feature importance ranking for the outpatient service utilization, which showed that the top 7 variables in the inpatient health services, with “frequency of diabetes medication use” and “household doctor” in the top two and “ethnicity” in the 7th. Figure 2b demonstrates the feature importance ranking for the inpatient service utilization, which also showed the top 7 variables in outpatient health services. In the model of inpatient service utilization, the “number of NCDs” variable obtained the highest score in this model, followed by the variable “self-assessment of health status”. The third important variable in this model is “medical insurance”, and the least important variable is “depression”.

Logistic regression analysis was performed on the factors affecting the utilization of outpatient services for people with diabetes in Gansu Province, using whether or not they visited a clinic within two weeks as the dependent variable, and using the forward stepwise LR method for variable screening (See Table 4). The inclusion and exclusion criteria were 0.05, 0.10, in that order. Logistic regression analysis showed that ethnicity, location, physical examination, anxiety or depression, and frequency of diabetes medication use had an impact on the utilization of outpatient services for diabetic patients. Minority patients were less likely to seek outpatient use than Han patients (OR = 0.172); patients with diabetes living in rural areas were 4.091 times more likely to seek outpatient use than those living in urban areas; patients without physical examination were less likely to seek outpatient use than those who underwent physical examination (OR = 0.317); patients with diabetes suffering from moderate to severe anxiety or depression were 0.370 times more likely to seek outpatient service use than patients with diabetes without anxiety or depression; and patients who occasionally used diabetes medication use (OR = 0.332) and never used diabetes medication use (OR = 0.200) were less likely to seek health services. Similarly, a logistic regression analysis was conducted to analyze the factors influencing the use of inpatient services among diabetic patients in Gansu Province, using a forward stepwise LR approach for variable screening with whether or not they were inpatient service within one year as the dependent variable. The results of the study demonstrated that health care insurance, physical pain and number of NCDs had an impact on whether patients with diabetes sought inpatient services. Patients with other types of health insurance (OR = 2.509) and no health insurance (OR = 5.305) were more likely to seek inpatient service than those with Urban Employees Basic Medical Insurance; patients with diabetes who had moderate to severe physical pain problems were 2.376 times more likely to seek inpatient service than those with no physical pain problems; in addition, it was observed that the likelihood of patients seeking inpatient service In addition, the likelihood of patients seeking inpatient services was observed to increase with the number of CHEs, with patients with one another chronic condition 1.198 times more likely to seek inpatient services than patients with diabetes alone, and patients with two other chronic conditions 2.538 times more likely to seek inpatient services than patients with diabetes alone.

**Table 4:** Results of logistic regression analysis of influencing factors

Variables	Reference category	*β*	Std	*Exp(B)*	*P*
*Outpatient health services*					
Ethnicity					
Minority	Han	− 1.759	0.644	0.172	0.006
Location	Urban				
Rural		1.409	0.354	4.091	< 0.001
Physical examination	Yes				
No		− 0.928	0.317	0.395	0.003
Anxiety or depression	No problems				
Medium or serious problems		1.040	0.370	2.829	0.005
Frequency of diabetes medication use	Regular dosing				
Occasionally		− 1.102	0.388	0.332	0.005
Never		− 1.612	0.704	0.200	0.022
*Inpatient health services*					
Health care insurance	UEBMI				
Other		0.920	0.441	2.509	0.037
None		1.669	0.928	5.305	0.072
Physical pain	No problems				
Medium or serious problems		0.865	0.361	2.376	0.017
Number of NCDs	1				
2		0.181	0.381	1.198	0.636
3 or more		0.931	0.433	2.538	0.032

**Fig. 3:**
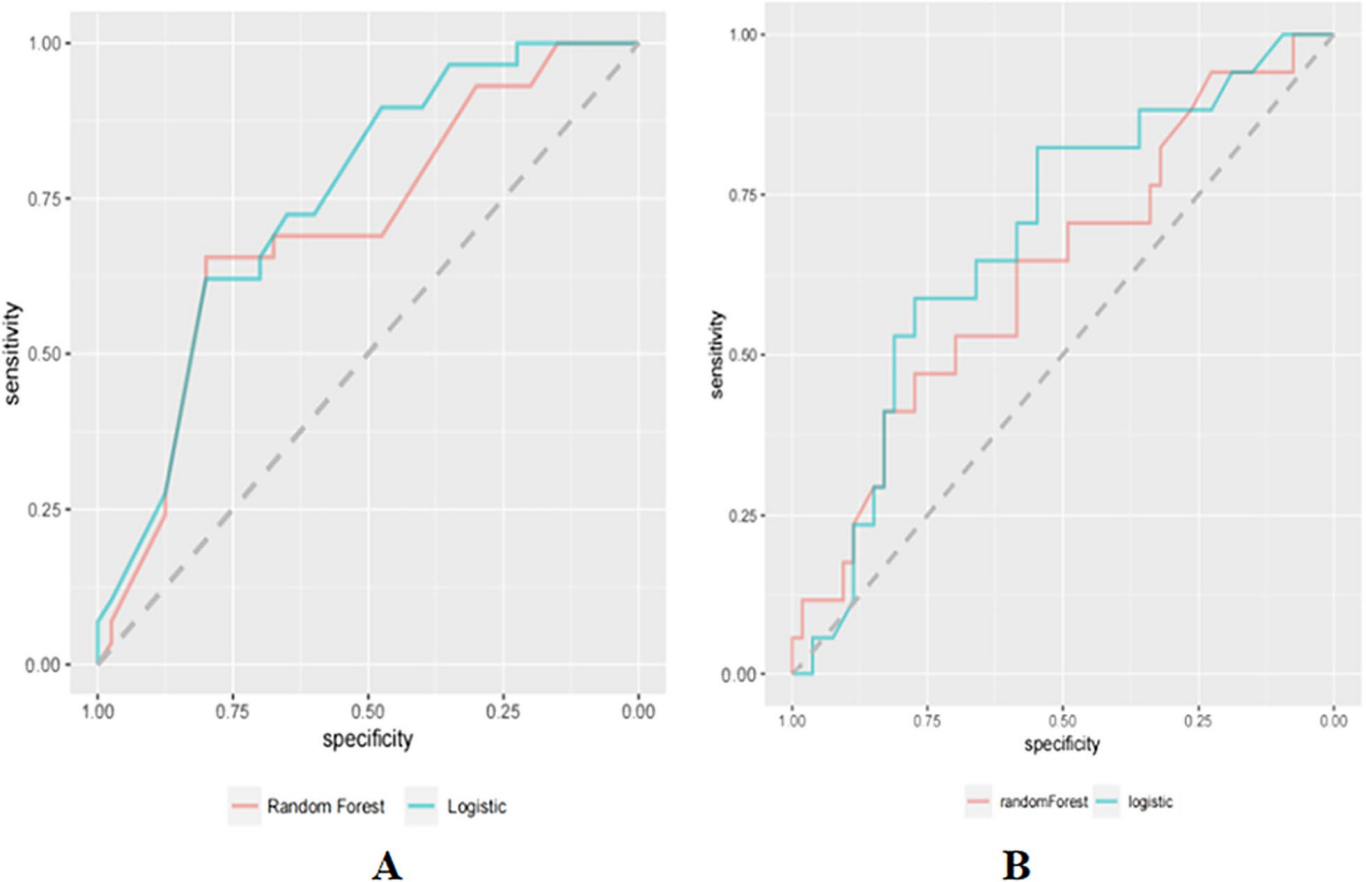
ROC analysis for health services: ROC analysis for outpatient health service (**A**), ROC analysis for inpatient health service (**B**)

The ROC comparison of the influencing factors affecting outpatient service utilization demonstrated that the area under the ROC curve was 0.706 (95% CI 0.581–0.831) in the random forest model and 0.748 (95% CI 0.634–0.863) in the logistic regression model, with the area under the ROC curve of the two models being close by Z-test and the difference was not statistically significant (See Fig. 3). A comparison of the ROCs for the influencing factors affecting inpatient service utilization revealed that the area under the ROC curve was 0.632 (95% CI 0.476–0.787) in the random forest model and 0.681 (95% CI 0.537–0.825) in the logistic regression model, with the area under the ROC curve of the two models being comparable by Z-test and the difference was not statistically significant.

### Equity analysis

The CIs for outpatient service utilization and inpatient service utilization were -0.241 and 0.107 for patients of different income levels, respectively, indicating that outpatient services were concentrated in patients of lower income levels and inpatient services tended to occur in patients of higher income levels (See Table 5). The CIs for both outpatient and inpatient service utilization for people with diabetes living in urban areas tended to occur in higher income patients at 0.149, 0.171, respectively. For people with diabetes living in rural areas, outpatient service utilization tended to occur in lower income patients (CI = − 0.229).

**Table 5:** Analysis of service utilization equity for people with diabetes

Variables	Wagstaff.CI	S.E	*P*
*Outpatient health service*			
Urban	0.149	0.163	0.363
Rural	− 0.229	0.079	0.004
Total	− 0.241	0.068	0.001
*Inpatient health service*			
Urban	0.171	0.139	0.220
Rural	− 0.017	0.099	0.862
Total	0.107	0.079	0.177

## Discussion

This study revealed the level of health service utilization of people with diabetes in Gansu Province, based on the framework of the Anderson Health Services Model, in addition to analyzing the main influencing factors on whether patients utilized health services.

Our study found that for factors affecting outpatient service utilization, frequency of diabetic medication was the most important factor affecting outpatient service utilization. Patients who used anti-diabetic drugs occasionally and never were 0.332 times and 0.200 times more likely to seek outpatient services than those who used anti-diabetic drugs regularly. Possible reasons for this were that health literacy among people with diabetes influenced self-care behavior, medication adherence and health-seeking behavior [21]. Therefore, patients on regular diabetic medication could manage their blood glucose by having good knowledge of diabetes and being able to actively seek relevant outpatient services. The result of the random forest revealed that whether or not a household doctor was contracted was a minor factor influencing patients to seek outpatient services. In China, household doctor services originated in 2009. In 2016, multiple departments jointly issued a document to further clarify the development goals of household doctor-contracted services in the next five years [22]. Contracted family doctors could meet the health needs of patients to some extent through health consultations and other means [23]. Similar studies have also shown that people living in rural areas are more likely to suffer from multiple NCDs than those living in towns, leading to a relatively higher number of visits to the doctor than the latter [24]. In addition, the location was the third most important factor influencing patients to seek outpatient services, and it was also found that patients living in rural areas were 4.091 times more likely to seek outpatient services than those living in urban areas. Although the random forest result showed that ethnicity was the least important factor, the logistic analysis showed that Han patients were more likely to seek outpatient services than minorities. There were relevant studies that showed that ethnicity had a significant impact on differences in health service utilization. Ethnic minority patients were less likely to use health services than Han Chinese patients, a finding consistent with a study conducted in Guangxi Province, China [25]. The possible reasons can be divided into two aspects: on the one hand, minorities rely mainly on relatives and friends to access social assistance network resources in urban communities [26]; on the other hand, religious beliefs could influence their health service-seeking behavior, and they might pray to God to relieve their suffering or take special herbal remedies to cure or alleviate their illness, rather than seeking health services [27]. Due to the small sample size of the minorities selected for this study, future research should be conducted in more depth on their health service utilization and the factors influencing it, using minorities as the target population.

The findings revealed that the top three factors that were most likely to influence people with diabetes to seek inpatient services were the number of NCDs, self-assessment of health status, and medical insurance. Also, the logistic results showed that people with diabetes who had a combination of 3 or more chronic conditions were 2.538 times more likely to seek inpatient services than those with only 1 chronic condition. Similar to previous studies, the higher the number of chronic disease categories, the higher the inpatient rate [9]. A study indicated that compared to Urban Employee Basic Medical Insurance (UEBMI) and Urban Resident Basic Medical Insurance (URBMI), in terms of inpatient rates, those enrolled in New Cooperative Medical Scheme (NCMS) had lower inpatient rates; in terms of actual hospital costs, they spent more than UEBMI participants because of lower reimbursement rates [28]. Differences in health insurance reimbursement rates might be the main reason for the differences in medical costs for diabetics with different health insurance [29]. Therefore, health insurance should be further integrated, and promoting health equity for patients, and strengthening financial protection for patients with chronic diseases. 

The finding revealed that outpatient service utilization was relatively equitable and that inequality in inpatient service utilization was significantly higher than outpatient service utilization. As diabetes is characterized by many complications, lifelong treatment, and poor prognosis, it can gradually impair the patient’s ability to work over a long period and gradually deplete the resources of the patient's family, thus reducing the living standards and wealth creation enabling of the patient's family [30]. First, the government should provide systematic economic risk protection for people with diabetes and other chronic diseases, especially low-income rural diabetics, such as exploring the establishment of a diabetes subsidy fund to play its role in providing medical assistance to low-income rural elderly diabetic families and fostering and guiding families to increase their income and wealth, to weaken the catastrophic effects of the disease and its tendency to inequality; Secondly, the health insurance benefit package should be further optimized and adjusted. Currently, China’s health insurance coverage is extensive, but the depth of coverage is limited, and the design of the health insurance benefit package does not focus on vulnerable groups such as elderly diabetics. Therefore, the design of the health insurance system should be further optimized to favor vulnerable groups such as the elderly with diabetes. Specifically, the proportion of health insurance reimbursement for people over 65 years of age and those with two or more NCDs should be further increased to reduce the risk of medical expenditures for families.

This study had some noteworthy strengths. Based on the Anderson Model of Health Services, this study used the random forest algorithm to explore the factors that may influence health service utilization and to analyze its equity between urban and rural areas. Random forests have several advantages over traditional impact factor analysis methods such as logistic regression. Firstly, ransom forests do not require a large sample size and can analyze data with influences larger than the sample size; secondly, random forests can analyze up to 32 categories of outcome variables. The two models can be used in combination when studying health services. These findings suggested the need for ongoing diabetes education for people with diabetes to address issues related to diabetes health literacy and adherence to medication, as well as to improve outpatient service utilization and better chronic disease management for people with diabetes [31]. However, the study also had some limitations. After a rigorous procedure of sampling, a sample size of 282 diabetic patients was finally selected for the study, which is slightly inadequate. In addition, the data used in this study lacked a timeline, so the findings were interpreted as correlations rather than causal effects. The following ideas will be expanded to explore changes in health service levels and associated influences across different timelines, etc. However, this study also has some limitations. The study used a random forest model approach to assess the importance of variables and logistic regression to analyze the likelihood of utilizing health services under different conditions, but the former may produce some bias when highly correlated characteristics are present. Despite the combined judgment in the interpretation of our results, this effect could not be completely ruled out. Secondly, the sample size was slightly inadequate for the survey and data processing under strict sampling procedures. In addition, the data used in this study was lack of timeline, so the findings were interpreted as associations rather than casual impact. Subsequent research will delve into more machine learning methods, select a larger sample size, and use patients with other chronic conditions as subjects to explore different timelines of health service utilization and analysis of influencing factors.

## Conclusion

This study described the level of outpatient services and inpatient services of people with diabetes in Gansu Province through relevant indicators, combining random forest and logistic regression analysis to seek the relevant factors that may influence whether or not patients used health services, identifying the factors that matter, and then presenting them in a ranked form in a chart. The study indicated that the top three factors that were likely to influence patients to seek outpatient services were frequency of diabetic medication, whether a household doctor had been contracted and their living location; the top three factors that were most likely to influence patients with diabetes to seek inpatient services were number of NCDs, self-assessment of health status, medical insurance. Outpatient services were focused on low-income groups and inpatient services were targeted at high-income groups. In the context of a universal health program, health is a basic human right, yet there are currently disparities in the utilization of health services across different residential settings and regions. The results of this study may help policymakers to prioritize the use of resources according to the order of importance, to achieve a rational use of medical services for diabetic patients, meet the medical service needs of different groups and allocate health resources rationally. In addition, under the premise of rational use of health services, medical coverage for NCDs patients should be appropriately increased and the financial burden of illness on the population should be reduced. Furthermore, the development of social medical assistance will ensure that every people suffering from diabetes has access to the most basic health services and achieve the goal of health for all.

## Data Availability

The datasets used and/or analyzed during the current study are available from the corresponding author on reasonable request.
